# Are men difficult to find? Identifying male-specific studies in MEDLINE and Embase

**DOI:** 10.1186/2046-4053-3-78

**Published:** 2014-07-18

**Authors:** Fiona Stewart, Cynthia Fraser, Clare Robertson, Alison Avenell, Daryll Archibald, Flora Douglas, Pat Hoddinott, Edwin van Teijlingen, Dwayne Boyers

**Affiliations:** 1 Health Services Research Unit, University of Aberdeen, Scotland, United Kingdom; 2 Centre of Academic Primary Care, University of Aberdeen, Aberdeen, Scotland; 3 Health Economics Research Unit, University of Aberdeen, Aberdeen, Scotland; 4 Rowett Institute of Health and Nutrition, University of Aberdeen, Aberdeen, Scotland; 5 Nursing, Midwifery and Allied Health Professional Research Unit, University of Stirling, Stirling, Scotland; 6 Centre for Midwifery, Maternal and Perinatal Health, Bournemouth University, Dorset, UK

**Keywords:** Bibliographic databases, Search filters, Information retrieval, Gender, Systematic reviews

## Abstract

**Background:**

Systematic reviews often investigate the effectiveness of interventions for one sex. However, identifying interventions with data presented according to the sex of study participants can be challenging due to suboptimal indexing in bibliographic databases and poor reporting in titles and abstracts. The purposes of this study were to develop a highly sensitive search filter to identify literature relevant to men's health and to assess the performance of a range of sex-specific search terms used individually and in various combinations.

**Methods:**

Comprehensive electronic searches were undertaken across a range of databases to inform a series of systematic reviews investigating obesity management for men. The included studies formed a reference standard set. A set of sex-specific search terms, identified from database-specific controlled vocabularies and from natural language used in the titles and abstracts of relevant papers, was investigated in MEDLINE and Embase. Sensitivity, precision, number needed to read (NNR) and percent reduction in results compared to searching without sex-specific terms were calculated.

**Results:**

The reference standard set comprised 57 papers in MEDLINE and 63 in Embase. Seven sex-specific search terms were identified. Searching without sex-specific terms returned 31,897 results in MEDLINE and 37,351 in Embase and identified 84% (MEDLINE) and 83% (Embase) of the reference standard sets. The best performing individual sex-specific term achieved 100%/98% sensitivity (MEDLINE/Embase), NNR 544/609 (MEDLINE/Embase) and reduced the number of results by 18%/17% (MEDLINE/Embase), relative to searching without sex-specific terms. The best performing filter, compromising different combinations of controlled vocabulary terms and natural language, achieved higher sensitivity (MEDLINE and Embase 100%), greater reduction in number of results (MEDLINE/Embase 24%/20%) and greater reduction in NNR (MEDLINE/Embase 506/578) than the best performing individual sex-specific term.

**Conclusions:**

The proposed MEDLINE and Embase filters achieved high sensitivity and a reduction in the number of search results and NNR, indicating that they are useful tools for efficient, comprehensive literature searching but their performance is partially dependent on the appropriate use of database controlled vocabularies and index terms.

## Background

Differences between the sexes often need to be taken into consideration in health services research. Notwithstanding sex-specific conditions and diseases, such as prostate cancer or pregnancy-related illnesses, the research questions of systematic reviews can often focus on one particular sex/gender so it is important to develop methods for efficient retrieval of relevant literature, with sufficient confidence in the comprehensiveness of the search methods. Furthermore, there is a growing body of published research evidence relating to sex/gender differences in non-sex-specific conditions and that these studies are difficult to identify in bibliographic databases [1].

In this report, we have used the terms ‘sex’ and ‘gender’ as defined by the World Health Organization:

‘The word ‘gender’ is used to define those characteristics of women and men that are socially constructed, while ‘sex’ refers to those that are biologically determined. People are born female or male but learn to be girls and boys who grow into women and men [2]’.

For brevity, we refer to both as ‘sex differences’ in this paper. There are sex differences in symptom presentation, prevalence and diagnosis of coronary heart disease [3] and rheumatoid arthritis. [4] Overweight and obesity are more prevalent in men in some industrialised countries such as the UK, while women are much more likely than men to engage in weight management interventions [5]. Sex can also affect how men and women use health services and how they are treated by health care professionals [6].

Identifying sex-specific evidence in the literature can be difficult due to suboptimal indexing in bibliographic databases, whereby index terms are not assigned in a way that is consistent with users' expectations. For instance, a paper entitled ‘Effectiveness of monetary contracts with two repayment schedules on weight reduction in men and women from self-referred and population samples’ [7] would not be retrieved in Embase by sex-specific controlled vocabulary terms, such as *Male/, Female/, Men/, Women/,* because it is not indexed with those terms. In such cases, relying on indexing in bibliographic databases is insufficient; consequently, natural language sex-specific search terms would be needed in order to identify relevant material. Alternatively, omitting such terms from search strategies that relate to sex-specific research questions would ensure comprehensiveness but is likely to yield unmanageable numbers of search results. Researchers conducting systematic reviews with a focus on a single sex would benefit from knowing which sex-specific search terms to use to identify literature relevant to men's/women's health within a practical timescale and without compromising the internal validity of the review.

Search filters made up of a combination of controlled vocabulary terms and natural language have been developed to identify collections of records with a common feature within bibliographic databases. Filters for specific study designs, such as randomised controlled trials (RCTs), diagnostic studies and economic evaluations, or for specific features of the population under consideration in a research question, such as age or geographic location, have been published and are widely used [8].

Reliable search filters are efficacious when conducting systematic reviews, especially where the condition or topic is broad, such as coronary heart disease or weight loss for obesity. Without filters, literature searches often produce impracticably high numbers of results which require an enormous amount of time spent by reviewers at the abstract screening stage. To the best of our knowledge, there are no pre-existing search filters designed to identify sex-specific literature related to adult men.

MEDLINE and Embase were selected as the most appropriate databases for testing search filters due to their pre-eminence in the health information retrieval field. Filters developed for MEDLINE and Embase can then be translated for use in other databases, although how well the filters perform in other databases is presently unknown.

The aim of this study was to develop a highly sensitive search filter to identify literature relevant to men's health by assessing the sensitivity, precision, reduction in number of results and number needed to read (NNR) of a range of sex-specific search terms used individually and in various combinations. In this context, we define sensitivity as the proportion of known relevant records identified by the search, precision as the proportion of records identified by the search that were relevant to the topic and NNR as the number of records screened per one relevant record identified.

## Methods

### Establishing a reference standard set

The performance of search filters should be tested against a ‘gold standard’ set of records, which should encompass all the known relevant records pertinent to the topic or theme under investigation. However, it is often impractical to determine the true gold standard set of studies; typically, a quasi-gold standard or reference standard is derived instead, which amounts to an approximation of the true gold standard. Authors of search filters have used a range of methods to compile reference standard sets [9].

Our reference standard set of records was derived from the studies included in a recent series of qualitative and quantitative systematic reviews on obesity management in men, the Review of MEn and Obesity (ROMEO) project [5]. The project systematically reviewed several aspects of obesity in men: the effectiveness and cost-effectiveness of interventions for obesity in men, the effectiveness of interventions to engage men in weight reduction and qualitative evidence from men in relation to obesity management. To be eligible for inclusion studies had to have either exclusively male participants or to report data separately for men and women.

Two information specialists (FS and CF) developed the search strategies for the ROMEO systematic reviews, which incorporated 18 bibliographic databases, 4 clinical trial registries and grey literature. The main searches were performed without sex-specific terms and were therefore suitable for establishing a reference standard set; however, for pragmatic reasons, i.e. to keep the number of search results within manageable parameters, some supplementary searches were performed with a male-specific focus. A team of reviewers independently screened around 15,000 titles and abstracts, with each record undergoing screening by at least two people. Additionally, studies were identified by contacting experts, commercial weight loss organisations and from the scrutiny of reference lists of relevant papers. The systematic and comprehensive methods used to identify studies ensured that the final set of included studies was as close as possible to representing the complete evidence base.

The title of each study included in the ROMEO project was searched for in two databases, MEDLINE and Embase, in order to calculate the proportion of those studies included in each. Two reference standard sets, one each for MEDLINE and Embase, comprised the ROMEO included studies that were identified by the ROMEO search strategies, i.e. the subject-only (SO) search, without sex-specific terms. The SO searches are provided in Additional file 1 and are also presented in full in the ROMEO final report [5].

All searches were carried out in March 2013 using the Ovid platform. The specific databases used were MEDLINE (1946 to 31 March 2013), MEDLINE-in-process and other non-indexed citations (31 March 2013) and Embase (1974 to 2013 week 13).

### Individual search terms

**Figure 1:**
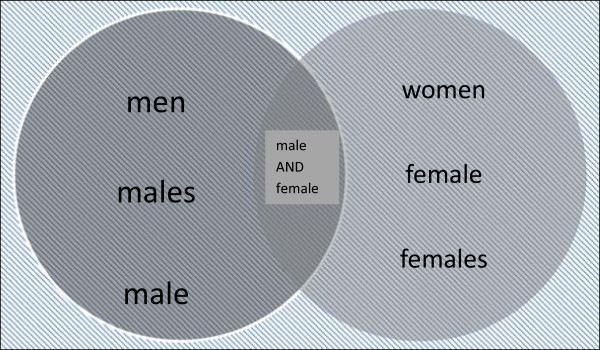
Venn diagram for NOTing out women.

A set of sex-specific search terms was identified from database-specific controlled vocabularies (MeSH and Emtree terms), by searching for male-related terms in the databases' permuted indexes, and from natural language used in the titles and abstracts of relevant papers. The candidate terms took three different approaches: some were designed to identify literature that explicitly referred to men, one was intended to exclude records indexed with the term *Female*/ and, finally, the others employed a double negative principle designed to exclude records explicitly referring to women but not to men. For clarification, a visual representation of NOTing out women-only material is given in Figure 1, which is a Venn diagram illustrating how we aimed to exclude all studies targeting women only, while also capturing those studies that reported results for women AND men. The candidate terms are designed to retrieve two finite sets of studies: a ‘male’ set and a ‘female’ set. The overlap between the two circles corresponds to the studies where results for both men and women are reported. Employing search terms designed to exclude studies featuring women, but not men, will therefore exclude studies corresponding to the right hand circle and will capture studies corresponding to the remaining three areas: the left hand circle, the overlap between the circles and the area falling outside either circle.

Each sex-specific search term under consideration (candidate term) was incorporated individually with the SO search, using the Boolean operator AND. For each candidate term, the sensitivity, precision, NNR and reduction in results relative to the SO search were calculated as follows:

$$\mathrm{Sensitivity}=\frac{\mathrm{Number}\;\mathrm{of}\;\mathrm{reference}\;\mathrm{standard}\;\mathrm{studies}\;\mathrm{identified}\;\mathrm{by}\;\mathrm{SO}\;\mathrm{AND}\;\mathrm{candidate}\;\mathrm{term}\times 100}{\mathrm{Total}\;\mathrm{number}\;\mathrm{of}\;\mathrm{reference}\;\mathrm{standard}\;\mathrm{studies}}$$

$$\mathrm{Precision}=\frac{\mathrm{Number}\;\mathrm{of}\;\mathrm{reference}\;\mathrm{standard}\;\mathrm{studies}\;\mathrm{by}\;\mathrm{SO}\;\mathrm{AND}\;\mathrm{candidate}\;\mathrm{term}\times 100}{\mathrm{Total}\;\mathrm{number}\;\mathrm{of}\;\mathrm{search}\;\mathrm{results}\;\mathrm{identified}\;\mathrm{SO}\;\mathrm{AND}\;\mathrm{candidate}\;\mathrm{term}}$$

$$\mathrm{Reduction}\;\mathrm{in}\;\mathrm{results}=\frac{\left(\mathrm{Total}\;\mathrm{number}\;\mathrm{of}\;\mathrm{results}\;\mathrm{identified}\;\mathrm{by}\;\mathrm{SO}\;\mathrm{search}-\left(\mathrm{Total}\;\mathrm{number}\;\mathrm{of}\;\mathrm{search}\;\mathrm{results}\;\mathrm{identified}\;\mathrm{by}\;\mathrm{SO}\;\mathrm{and}\;\mathrm{term}\times 100\right)\right)}{\mathrm{Total}\;\mathrm{number}\;\mathrm{of}\;\mathrm{search}\;\mathrm{results}\;\mathrm{identified}\;\mathrm{by}\;\mathrm{SO}\;\mathrm{search}}$$

The performance of each candidate term was evaluated by the extent to which it reduced the NNR and number of results relative to the SO search as well as the extent to which sensitivity was maintained and precision increased. Because the filter was developed with systematic review methods in mind, sensitivity was prioritised over reduction in results; for instance, a candidate term that reduced the number of results by 50% had a lower NNR than the SO search but achieved 60% sensitivity would not be considered as effective as a candidate term that reduced the number of results by 20% and achieved 90% sensitivity. The individual candidate term with the best balance between sensitivity and reduction in results, as assessed by two authors (FS and CF), was used as a benchmark against which to test the performance of combined sets of candidate terms.

### Candidate filters: combining candidate terms

All candidate terms, combined with the Boolean operator OR, were incorporated with the SO search, using the Boolean operator AND. Sensitivity, reduction in results and NNR were calculated for the full set of candidate terms incorporated with the SO search. This process was then repeated for all possible combinations of two or more candidate terms and the results were compared to the best performing individual candidate term.

### Validating the filters

To obtain further evidence of the performance of the search filters, they should be tested against an independently derived reference set of records obtained from search strategies designed to identify male-specific studies but which did not include sex-specific search terms. Searches were undertaken in the Cochrane Database of Systematic Reviews and the Database of Abstracts of Reviews of Effects to identify an appropriate validation set, which would ideally come from existing systematic reviews, pertaining to an aspect of men's health (but not to a sex-specific condition), and which also does not include male-specific terms in its search strategy. Sex-specific conditions such as prostate cancer would not be suitable because sex specificity is implicit in the condition itself; therefore, the use of a sex-specific filter would be superfluous.

Upon identification of a suitable systematic review with an appropriate search strategy, the filters would be tested by ascertaining the proportion of the review's included studies that are identified by its original search strategy combined with AND with each filter in turn.

## Results

### Description of reference standard

The number of included studies in the ROMEO project was 87, of which 57 (66%) were indexed in MEDLINE and 63 (72%) in Embase. Fifty-three studies (61%) were indexed by both MEDLINE and Embase and 20 were not in either database. Of the 57 studies indexed by MEDLINE, 48 (84%) were identified by the SO search, without sex-specific search terms, while 52 (83%) of the 63 studies indexed by Embase were identified by the SO search, without sex-specific search terms; therefore, the reference standard sets comprised 48 studies (MEDLINE) and 52 studies (Embase). Five studies (9%) were not identified by the SO search in either database. The NNR was 665 in MEDLINE and 718 in Embase.

### Candidate terms identified

Eight sex-specific candidate search terms were identified and incorporated with the SO search (Table 1). Three of the candidate terms were made up of natural language and five were controlled vocabulary terms. All five controlled vocabulary terms are used as both MeSH and Emtree terms.

**Table 1:** Candidate search terms

**Search number**	**Search term**
S1	[SO] NOT ((women not men) OR (female not male)).tw
S2	[SO] NOT ((women or female) NOT (men or male)).tw
S3	[SO] AND (male or males or men).tw
S4	[SO] NOT Female/
S5	[SO] AND Male/
S6	[SO] NOT (Female/not Male/)
S7	[SO] AND Men/
S8	[SO] AND Men's health/

*SO* subject-only search, */* MeSH term or Emtree term, *.tw* text word(s) from title and/or abstract fields.

### Performance of individual candidate terms

**Figure 2:**
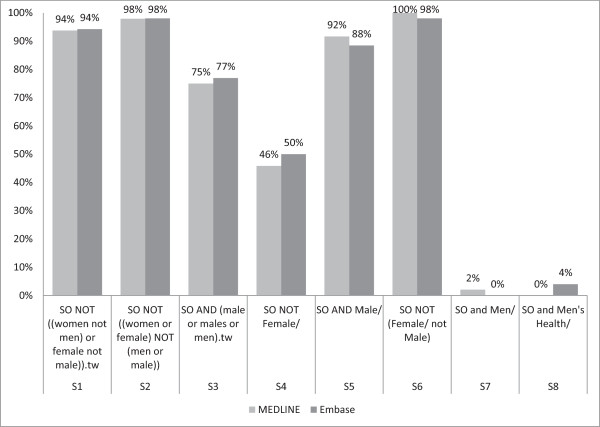
Individual candidate terms: sensitivity.

**Figure 3:**
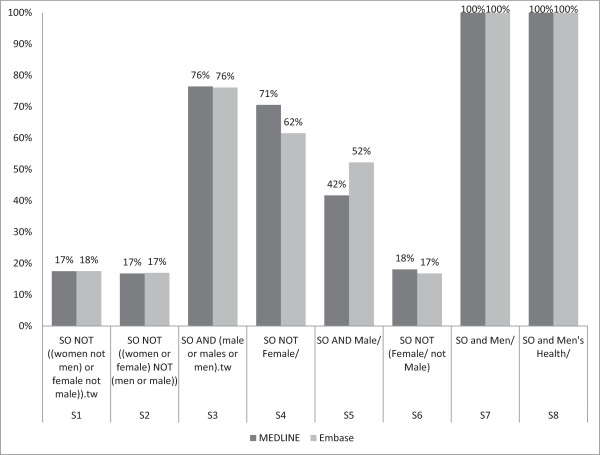
Individual candidate terms: percent reduction in results relative to SO search.

The sensitivity of the individual candidate terms ranged from 0% to 100% (Figure 2), where the terms with 0% sensitivity returned none of the reference standard set of studies, and the reduction in results compared to the SO search ranged from 17% to 100% (Figure 3). Precision ranged from 0.17% to 16.67% (median 0.23%). With the exception of the outlier terms S7 (*Men/*) and S8 (*Men's health/)*, NNR ranged from 209–585 (MEDLINE) and 223–629 (Embase) (Table 2). For sensitivity, reduction in results and NNR, there were no substantial differences between the performances of the two databases, again with the notable exception of S7 (*Men/*).

**Table 2:** Total number of results and NNR of individual terms

	**Term**	**MEDLINE**				**Embase**			
		**Total results**	**NNR**	**Sensitivity (*n *** **= 48)**	**Precision**	**Total results**	**NNR**	**Sensitivity (*n *** **= 52)**	**Precision**
SO	Subject-only search	31,897	665	100% (48)	0.15%	37351	718	100%	0.14%
S1	[SO] NOT ((women NOT men) OR (female NOT male)).tw	26,318	585	94% (45)	0.17%	30801	629	94% (49)	0.16%
S2	[SO] NOT ((women OR female) NOT (men or male)).tw	26,544	565	98% (47)	0.18%	31011	608	98% (51)	0.16%
S3	[SO] AND (male or males or men).tw	7,513	209	76% (36)	0.48%	8921	223	76% (40)	0.45%
S4	[SO] NOT Female/	9,385	427	71% (22)	0.23%	14362	552	62% (26)	0.18%
S5	[SO] AND Male/	18,599	423	42% (44)	0.24%	17848	388	52% (46)	0.26%
S6	[SO] NOT (Female/NOT Male/)	26,127	544	100% (48)	0.18%	31082	609	98% (51)	0.16%
S7	[SO] AND Men/	6	6	2% (1)	16.67%	N/A	N/A	N/A	N/A
S8	[SO] AND Men's health/	7	N/A	0% (0)	N/A	16	8	4% (2)	12.50%

Term S6 [*NOT (Female/not Male/)*] was judged to have achieved the best performance with 100% and 98% sensitivity in MEDLINE and Embase, respectively, as well as a reduction in total number of results by 5,770 (18%) in MEDLINE and 6,269 (17%) in Embase and an NNR of 544 (MEDLINE) and 609 (Embase).

### Performance of candidate filters

Due to their outlying performances as individual candidate terms, S7 (*Men/*) and S8 (*Men's health/*) were removed from the combinations of search terms. Precision ranged from 0.16% to 0.24% (median 0.18%). Most combinations of search terms achieved 100% sensitivity in both MEDLINE and Embase (Table 3). Terms S3 (*male or males or men.tw*) and S4 (*NOT Female/*) combined with OR, and incorporated with the SO search with AND, achieved the biggest reduction in results compared to the SO search, reducing results by 19,508 (61%) in MEDLINE and 16,757 (45%) in Embase, but this combination had the lowest sensitivity of all the combinations tested, achieving 79% (MEDLINE) and 85% (Embase).

**Table 3:** Candidate filters: total number of results, NNR and sensitivity

	**MEDLINE**					**Embase**				
	**Total results**	**NNR**	**Sensitivity (*n *** **= 48)**	**% reduction in results**	**Precision**	**Total results**	**NNR**	**Sensitivity (*n *** **= 52)**	**% reduction in results**	**Precision**
SO	31897	665	100% (48)		0.15%	37351	718	100% (**52)**		0.14%
[SO] AND (***S1 OR S5)***_***1***_	***24277***	***506***	***100%*** (48)	***24%***	***0.20%***	***30046***	***578***	***100%*** (**52)**	***20%***	***0.17%***
[SO] AND (S1 OR S3 OR S5)_***2***_	24324	507	100% (48)	24%	0.20%	30089	579	100% (52)	19%	0.17%
[SO] AND (S3 OR S4 OR S5)	24207	504	100% (48)	24%	0.20%	32136	618	100% (52)	14%	0.16%
[SO] AND (S1 OR S3 OR S4 OR S5)	25874	539	100% (48)	19%	0.19%	30389	584	100% (52)	19%	0.17%
[SO] AND (S1 OR S2 OR S5)	25796	537	100% (48)	19%	0.19%	30076	578	100% (52)	19%	0.17%
[SO] AND (S1 OR S2 OR S3 OR S4)	25864	539	100% (48)	19%	0.19%	32352	622	100% (52)	13%	0.16%
[SO] AND (S1 OR S2 OR S4 OR S5)	25874	539	100% (48)	19%	0.19%	32409	623	100% (52)	13%	0.16%
[SO] AND (S1 OR S2 OR S3 OR S4 OR S5)	26028	542	100% (48)	18%	0.18%	33235	639	100% (52)	11%	0.16%
[SO] AND (S2 OR S3 OR S4 OR S5)	26028	542	100% (48)	18%	0.18%	33235	639	100% (52)	11%	0.16%
[SO] AND (S4 OR S5)	24137	503	100% (48)	24%	0.20%	31860	625	98% (51)	15%	0.16%
[SO] AND (S1 OR S2 OR S3 OR S5)	25806	549	98% (47)	19%	0.18%	31925	614	100% (52)	14%	0.16%
[SO] AND (S1 OR S2 OR S4)	24808	528	98% (47)	22%	0.19%	29432	577	98% (51)	21%	0.17%
[SO] AND (***S3 OR S5)***	***20388***	***425***	***100%*** (48)	***36%***	***0.24%***	***21367***	***427***	***96% (50)***	***43%***	***0.23%***
[SO] AND (S1 OR S4)	23543	490	100% (48)	26%	0.20%	29233	585	96% (50)	22%	0.17%
[SO] AND (S1 OR S3)	23372	497	98% (47)	27%	0.20%	29226	573	98% (51)	22%	0.17%
[SO] AND (S1 OR S2 OR S3)	24820	528	98%(47)	22%	0.19%	29226	573	98% (51)	22%	0.17%
[SO] AND (S1 OR S2)	25241	537	98% (47)	21%	0.19%	31807	624	98% (51)	15%	0.16%
[SO] AND (S3 OR S4)	12389	326	79% (38)	61%	0.31%	20604	468	85% (44)	45%	0.21%

1 filter A.

2 filter B.

The reduction in number of results ranged from 18%-61% (MEDLINE) and 11%-45% (Embase) and the NNR ranged from 326–549 (MEDLINE) and 427–639 (Embase). Full details of the sensitivity and NNR are presented in Table 3. The majority of combinations achieved 100% sensitivity, with the combination S1 OR S5 (hereafter denoted filter A, highlighted in bold italics in Table 3) attaining the biggest reduction in results (24%/20% in MEDLINE/Embase). Filter A's NNR when compared to searching without sex-specific terms was 506/578 (MEDLINE/Embase).

Filter A was therefore judged to be the most appropriate set of terms to use when designing a search for maximum sensitivity. The terms included in filter A were as follows:

1. SO search

2. 1 NOT ((women NOT men) OR (female NOT male)).tw

3. 1 AND Male/

4. or/2-3

While filter A reduced the number of search results by almost a quarter, one other filter achieved a greater reduction in results while also approaching 100% sensitivity. S3 OR S5 (hereafter denoted as filter B, highlighted in bold italics in Table 3) achieved 36% and 43% fewer results in MEDLINE and Embase, respectively, compared with searching without sex-specific terms. Filter B also achieved a substantially lower NNR than searching without sex-specific terms (filter B NNR = 425/427, MEDLINE/Embase). While 100% sensitivity was reached in MEDLINE, two of the reference standard sets were not picked up in Embase, one of which was identified in MEDLINE but the remaining article is not indexed in MEDLINE and therefore would not be identified at all. The terms included in filter B were as follows:

1. SO search

2. 1 AND (male or males or men).tw

3. 1 AND Male/

4. or/2-3

### Validation

The searches conducted to find an appropriate validation standard did not find any suitable reviews. The main reasons for this were that the search strategies used either were not reported in full, already included sex-specific terms or were run in non-Ovid databases and therefore could not be translated. Consequently, it is not yet possible to test the filters against external standards.

## Discussion

In answering the question posed in this paper, our results suggest that when searching for sex-specific literature, men are difficult to find without incorporating women into the search strategy. This is important when the aim is to conduct comprehensive, systematic searches designed to identify all relevant material pertinent to a sex-specific research question. The 100% sensitivity of filter A indicates that searches using the Boolean operator NOT to exclude records that mention women, but not men, is more effective than restricting searches with the use of male-specific terms. Filter A is a highly sensitive method of identifying literature relevant to men's health, which substantially reduces the number of results compared to searching without sex-specific terms. However, filter B achieves very close to 100% sensitivity with a greater reduction in results than filter A and uses simple male-specific search terms. The lower NNR of filter B indicates its greater potential for saving time when screening search results, compared to filter A. Nevertheless, to maintain confidence in a search's sensitivity, for instance in the context of conducting a systematic review, filter A is preferable.

### Reference standard sets

Five of the ROMEO included studies were indexed in MEDLINE and Embase but were not identified by the SO search in either database, having been identified by hand-searching for inclusion in the ROMEO project, and were therefore not included in the reference standard sets. However, all five studies were indexed with *Male/* and would have been picked up by either filter A or B. The reasons for their omission from the SO search were attributed to the terms included in the SO search; for instance, one study was not picked up because it did not include any index terms or text words relating to the weight loss facet of the SO search.

### Agreement between MEDLINE and Embase

The similarity between MEDLINE and Embase, in terms of sensitivity and reducing the number of results, supports the appropriateness of using the same filter in both databases without any requirement for translation from one database to the other. However, NNR was consistently higher in Embase than in MEDLINE, which is perhaps explained by the wider coverage of Embase, which contains over 28 million records [10] compared to MEDLINE's 20 million [11]. Furthermore, the differing approaches to indexing in MEDLINE and Embase could also have had an impact on NNR. MEDLINE's indexing guide stipulates that records with more than three non-major concepts will be indexed with general rather than specific MeSH terms [12], whereas Embase records can be indexed with up to 50 minor terms [13]. Embase's wider coverage and indexing policy mean that the same index term is likely to identify more records in Embase than in MEDLINE.

### Controlled vocabulary terms

All five controlled vocabulary terms considered for the filters are used in both MEDLINE and Embase, which means that, unlike many existing search filters [8], these can be used without any need for translation from one database to another.

However, the controlled vocabulary term *Men/* is used differently in MEDLINE and Embase. As a MeSH term in MEDLINE, it is intended for use in the context of ‘men or boys only as a cultural, social, sociological, political, economic force’ [14], distinct from male as a biological sex, while in Embase *Men/* maps directly to the term *Male/* and therefore *Men/* and *Male/* retrieve exactly the same set of Embase records. *Men/* as a MeSH term is used in MEDLINE only 2,560 times in its 20 million records (as of January 2014). A substantial proportion of our reference standard set comprised qualitative studies relating to sex differences in perceptions of obesity and weight loss, i.e. men as a ‘cultural force’ contrasted with women; however, only one study from the reference standard set was identified by *Men/*.

The controlled vocabulary term *Men's health/* was similarly problematic. While it is listed as an index term in both the MeSH and Emtree thesauri, its use extends to only 1,719 Embase records and 1,071 MEDLINE records (as of January 2014). Two of the Embase reference standard studies and none of the MEDLINE reference standard set were identified by the SO search AND *Men's health/*. The MEDLINE scope notes indicate that *Men's health/* relates to ‘the concept covering the physical and mental conditions of men’ [15]; this wide-ranging definition could reasonably be expected to be applicable to a large body of literature. *Men's health* was introduced to the controlled vocabularies relatively recently (Embase in 2006 and MEDLINE in 2008), which may partly explain the low number of records, but considering that the majority of the reference standard set of articles were published since 2006, and that many of them have a distinct focus on men's health, it is perhaps surprising that the term *Men's health/* has not been used more frequently. Sex differences are increasingly taken into account in health services research, as evidenced by the establishment of initiatives such as the Campbell and Cochrane Equity Methods Group's Working Group on ‘Sex and Gender Analysis in Systematic Reviews’. It would be beneficial for searchers to have confidence that terms such as *Men/* and *Men's health/* will identify relevant records.

The relatively high sensitivity of the index term *Male/* indicates that searchers can use that term with confidence when searching for literature relevant to men's health, but combining *Male/* with natural language terms, with the Boolean operator OR, is required to achieve optimum sensitivity. The lower sensitivity of *Male/* used by itself, without combining with natural language terms, is most likely explained by the time lag between a record's date of entry to the database and the assignment of index terms, which can often take weeks or months.

### Precision

Precision is one of the key indicators in assessing the performance of a search or search filter. The precision of the search filters tested here was considerably below 3%, the median value found by Sampson and colleague's cross-sectional study of 94 systematic review search strategies [16]. The low precision of our filters is partly attributable to the design of the SO search, which was intended to identify literature relating to the broad topics of obesity and weight loss. It is likely that a more specific and narrowly defined subject area would result in higher precision. Nevertheless, the increases in precision from 0.15%/0.14% to 0.20%/0.17% in MEDLINE/Embase when using filter A and from 0.15%/0.14% to 0.24%/0.24% when using filter B represent considerable advantages to be gained from using the filters.

### Limitations of the study and implications for further research

We recognise that the filters have not been tested against a true gold standard set of studies, which is typically achieved by hand-searching a set of pre-specified journals. However, we are confident that the methods adopted to derive the reference standard sets used here were robust and systematic and therefore resulted in an appropriate and reliable reference standard against which to test candidate terms for the sex-specific filter. Nevertheless, we acknowledge that the reference standard used here is limited to a relatively small number of studies and that our findings essentially represent a single case study and may not be generalisable.We also recognise that the performance of the filters has not been assessed against external validation standards, i.e. tested against independently derived search strategies designed to identify male-specific studies but which did not include sex-specific search terms. Further research may be needed to determine if the filters we have developed for MEDLINE and Embase are applicable to other bibliographic databases. The principle of NOTing out one sex, as illustrated in Figure 1, may require further testing in other databases. Furthermore, it remains to be seen whether optimum search methods for identifying literature relevant to women's health will entail NOTing out men in a similar way.

The filters we have developed relate to men as a biological sex. Further research could explore the development of filters for people who self-identify as men but who are not biologically male, or who self-identify as women but are not biologically female.

## Conclusions

We have demonstrated that the suggested sex-specific filters, A and B, are suitable for use in MEDLINE and Embase. The filters maintain the sensitivity of the original subject search, while reducing the number of search results to be screened by 20%–43%. For systematic reviewers undertaking literature searches relating to men's health, where the health condition or disease is not sex-specific, utilising filter A or B will be beneficial in substantially reducing the number of records to screen. The choice of filter will likely depend on the time available for the searching and screening processes. Further exploration is desirable to test the filters with alternative datasets and to adapt them for use in other databases.

## Competing interests

The authors declare that they have no competing interests.

## Authors' contributions

FS developed and tested the filters and drafted the manuscript with advice and supervision from CF. AA oversaw and coordinated all aspects of the ROMEO project. CR, DA and DB screened the records and carried out analysis for the ROMEO project. FD, PH and EvT co-supervised data analysis for the ROMEO project. All authors contributed to the study design, manuscript preparation and revisions. All authors read and approved the final manuscript.

## Supplementary Material

Additional file 1 Search strategy. Click here for file
