# Supplementary material for: Are men difficult to find? Identifying male-specific studies in MEDLINE and Embase
Source: Syst Rev. 2014 Jul 18;3:78. doi: 10.1186/2046-4053-3-78 (PMC4120011; doi:10.1186/2046-4053-3-78)
Supplement: Additional file 1 — Search strategy. [file 2046-4053-3-78-S1.docx]

**Additional file 1 Appendix 1 Subject-only (SO) search**

**Embase (1974 to 31^st^ March 2013)**

1. obesity/
2. (obesity adj2 (morbid or diabet$)).tw.
3. morbid obesity/
4. obes$.tw.
5. weight reduction/
6. (weight adj1 (los$ or reduc$ or maint$ or control or manag$)).tw.
7. (diet adj5 weight).tw.
8. overweight.tw.
9. (obesity adj1 management).tw.
10. (anti obesity or antiobesity).tw.
11. or/1-10
12. *obesity/
13. obesity hypoventilation syndrome/
14. abdominal obesity/
15. diabetic obesity/
16. (overweight or obes$).ti.
17. (obes$ adj1 (morbid or diabet$ or abdom$ or central)).tw.
18. or/3,12-17
19. *weight reduction/
20. (weight adj1 (los$ or reduc$ or maint$ or control or manag$)).tw.
21. (reduc$ adj2 (waist adj3 (ratio or circumference))).tw.
22. (reduc$ adj2 (bmi or body mass index)).tw.
23. anti obesity.tw.
24. (obesity adj1 manag$).tw.
25. or/19-24
26. or/12-24
27. randomization/
28. exp clinical trial/
29. Randomized Controlled Trials as Topic/
30. randomi?ed.ab.
31. placebo.ab.
32. randomly.ab.
33. trial.ab.
34. groups.ab.
35. or/27-34
36. qualitative research/
37. exp questionnaire/
38. exp interview/
39. (qualitative or interview$ or focus group$ or questionnaire$ or survey$).tw.
40. (ethno$ or grounded or thematic or interpretive or narrative or realist$ or meta stud$ or experience?).tw.
41. or/36-40
42. treatment outcome/
43. controlled study/
44. clinical trial/
45. (preoperat$ or pre operat$).tw.
46. (chang$ or evaluat$ or reviewed or baseline).tw.
47. ((prospective$ or retrospective$) adj1 (study or studies)).tw.
48. (cohort$ or case series).tw.
49. ((compare$ or compara$) adj1 (study or studies)).tw.
50. or/42-49
51. *"patient acceptance of health care"/ or *patient compliance/ or *medication adherence/ or *patient participation/ or exp *patient satisfaction/ or treatment refusal/
52. exp health promotion/
53. *patient dropouts/
54. exp patient compliance/
55. exp Consumer Participation/
56. (uptake or retention or retain or engag$ or particip$ or motivat$ or encourag$ or attrition or dropout or promot$ or recruit$ or involv$).tw.
57. or/51-56
58. ((male or men?) adj3 (uptake or retention or retain or engag$ or particip$ or motivat$ or encourag$ or attrition or dropout or promot$ or recruit$ or involv$)).tw.
59. ((service$ or program$ or scheme$ or initiative$ or intervention$ or diet$) adj3 ((uptake or retention or retain or engag$ or particip$ or motivat$ or encourag$ or attrition or dropout or promot$ or recruit$ or involv$) adj3 (male or men?))).tw.
60. ((uptake or retention or retain or engag$ or particip$ or motivat$ or encourag$ or attrition or dropout or promot$ or recruit$ or involv$) adj3 (men? or male) adj3 ((obesity adj1 manag$) or (weight adj1 (los$ or reduc$ or maint$ or control or manag$)) or (overweight or obes$))).tw.
61. 57 and 60
62. 26 and 58
63. 26 and 59
64. or/61-63
65. exp great britain/
66. (united kingdom or uk or britain or scotland or england or wales or northern ireland or british or irish or scottish or welsh or english).tw.
67. (United kingdom or uk or Britain or scotland or England or wales or Ireland or London or Birmingham or Leeds or Glasgow or Sheffield or Bradford or Edinburgh or Liverpool or Manchester or Bristol or Wakefield or Cardiff or Coventry or Nottingham or Leicester or Sunderland or Belfast or Newcastle upon Tyne or Brighton or Hull or Plymouth or Stoke-on-Trent or Wolverhampton or Derby or Swansea or Southampton or Salford or Aberdeen or Westminster or Portsmouth or York or Peterborough or Dundee or Lancaster or Oxford or Newport or Preston or Norwich or Chester or Cambridge or Salisbury or Exeter or Gloucester or Lisburn or Chichester or Winchester or Londonderry or Carlisle or Worcester or Bath or Durham or Lincoln or Hereford or Armagh or Inverness or Stirling or Canterbury or Lichfield or Newry or Ripon or Bangor or Truro or Ely or Wells).in.
68. or/65-67
69. 11 and 35
70. 18 and 25 and 41
71. 18 and 25 and 50 and 68
72. or/64,69-71
73. exp animals/ not humans/
74. (comment$ or letter or editorial or review or case report).pt.
75. limit 72 to (embryo or infant or child or preschool child <1 to 6 years> or school child <7 to 12 years> or adolescent <13 to 17 years>)
76. 72 not (73 or 74 or 75)

**MEDLINE (1946 to 31^st^ March 2013)**

**MEDLINE-in-Process and Other Non-Indexed Citations (31^st^ March 2013**

1. obesity/
2. (obesity adj2 (morbid or diabet$)).tw.
3. obesity, morbid/
4. obes$.tw.
5. weight loss/
6. (weight adj1 (los$ or reduc$ or maint$ or control or manag$)).tw.
7. (diet adj5 weight).tw.
8. overweight.tw.
9. (obesity adj1 management).tw.
10. (anti obesity or antiobesity).tw.
11. or/1-10
12. *obesity/
13. obesity hypoventilation syndrome/
14. obesity, abdominal/
15. (overweight or obes$).ti.
16. (obes$ adj1 (morbid or diabet$ or abdom$ or central)).tw.
17. or/12-16
18. *weight loss/
19. (weight adj1 (los$ or reduc$ or maint$ or control or manag$)).tw.
20. (reduc$ adj2 (waist adj3 (ratio or circumference))).tw.
21. (reduc$ adj2 (bmi or body mass index)).tw.
22. anti obesity.tw.
23. (obesity adj1 manag$).tw.
24. or/18-23
25. or/12-23
26. exp clinical trial/
27. Randomized Controlled Trials as Topic/
28. randomized controlled trial.pt.
29. controlled clinical trial.pt.
30. randomi?ed.ab.
31. placebo.ab.
32. drug therapy.fs.
33. randomly.ab.
34. trial.ab.
35. groups.ab.
36. or/26-35
37. qualitative research/
38. exp questionnaires/ use prmz
39. exp interviews as topic/ use prmz
40. (qualitative or interview$ or focus group$ or questionnaire$ or survey$).tw.
41. (ethno$ or grounded or thematic or interpretive or narrative or realist$ or meta stud$ or experience?).tw.
42. or/37-41
43. comparative study/ use prmz
44. follow-up studies/ use prmz
45. time factors/ use prmz
46. (preoperat$ or pre operat$).tw.
47. (chang$ or evaluat$ or reviewed or baseline).tw.
48. ((prospective$ or retrospective$) adj1 (study or studies)).tw.
49. (cohort$ or case series).tw.
50. ((compare$ or compara$) adj1 (study or studies)).tw.
51. or/43-50
52. *"patient acceptance of health care"/ or *patient compliance/ or *medication adherence/ or *patient participation/ or exp *patient satisfaction/ or treatment refusal/
53. exp health promotion/
54. *patient dropouts/
55. exp patient compliance/
56. exp Consumer Participation/
57. (uptake or retention or retain or engag$ or particip$ or motivat$ or encourag$ or attrition or dropout or promot$ or recruit$ or involv$).tw.
58. or/52-57
59. ((male or men?) adj3 (uptake or retention or retain or engag$ or particip$ or motivat$ or encourag$ or attrition or dropout or promot$ or recruit$ or involv$)).tw.
60. ((service$ or program$ or scheme$ or initiative$ or intervention$ or diet$) adj3 ((uptake or retention or retain or engag$ or particip$ or motivat$ or encourag$ or attrition or dropout or promot$ or recruit$ or involv$) adj3 (male or men?))).tw.
61. ((uptake or retention or retain or engag$ or particip$ or motivat$ or encourag$ or attrition or dropout or promot$ or recruit$ or involv$) adj3 (men? or male) adj3 ((obesity adj1 manag$) or (weight adj1 (los$ or reduc$ or maint$ or control or manag$)) or (overweight or obes$))).tw.
62. 58 and 61
63. 25 and 59
64. 25 and 60
65. or/62-64
66. exp great britain/
67. (united kingdom or uk or britain or scotland or england or wales or northern ireland or british or irish or scottish or welsh or english).tw.
68. (United kingdom or uk or Britain or scotland or England or wales or Ireland or London or Birmingham or Leeds or Glasgow or Sheffield or Bradford or Edinburgh or Liverpool or Manchester or Bristol or Wakefield or Cardiff or Coventry or Nottingham or Leicester or Sunderland or Belfast or Newcastle upon Tyne or Brighton or Hull or Plymouth or Stoke-on-Trent or Wolverhampton or Derby or Swansea or Southampton or Salford or Aberdeen or Westminster or Portsmouth or York or Peterborough or Dundee or Lancaster or Oxford or Newport or Preston or Norwich or Chester or Cambridge or Salisbury or Exeter or Gloucester or Lisburn or Chichester or Winchester or Londonderry or Carlisle or Worcester or Bath or Durham or Lincoln or Hereford or Armagh or Inverness or Stirling or Canterbury or Lichfield or Newry or Ripon or Bangor or Truro or Ely or Wells).in.
69. or/66-68
70. 11 and 36
71. 17 and 24 and 42
72. 17 and 24 and 51 and 69
73. or/65,70-72
74. exp animals/ not humans/
75. limit 73 to ("all infant (birth to 23 months)" or "all child (0 to 18 years)")
76. (comment$ or letter or editorial or review or case report).pt.
77. 73 not (74 or 75 or 76)

**Key**

/ = MeSH or Emtree term

.tw = textword

.ab = abstract

.fs = floating subheading

pt = publication type

exp = explode MeSH or Emtree term

adj1 = adjacency operator

$ = truncation

? = wildcard

* = focus MeSH or Emtree term

.
